# Curcumin Enhances Fed-State Muscle Microvascular Perfusion but Not Leg Glucose Uptake in Older Adults

**DOI:** 10.3390/nu14061313

**Published:** 2022-03-21

**Authors:** Colleen S. Deane, Ushnah S. U. Din, Tanvir S. Sian, Ken Smith, Amanda Gates, Jonathan N. Lund, John P. Williams, Ricardo Rueda, Suzette L. Pereira, Philip J. Atherton, Bethan E. Phillips

**Affiliations:** 1 Department of Sport and Health Sciences, College of Life and Environmental Sciences, University of Exeter, Exeter EX1 2LU, UK; c.s.deane@exeter.ac.uk; 2 Living Systems Institute, University of Exeter, Stocker Road, Exeter EX4 4QD, UK; 3 MRC-Versus Arthritis Centre for Musculoskeletal Ageing Research and National Institute for Health Research Nottingham Biomedical Research Centre, School of Medicine, University of Nottingham, Derby DE22 3DT, UK; ushnah.din@nottingham.ac.uk (U.S.U.D.); tanvirsian@gmail.com (T.S.S.); ken.smith@nottingham.ac.uk (K.S.); amanda.gates@nottingham.ac.uk (A.G.); jon.lund@nottingham.ac.uk (J.N.L.); john.williams7@nottingham.ac.uk (J.P.W.); 4 Department of Surgery and Anaesthetics, Royal Derby Hospital, Derby DE22 3NE, UK; 5 Research and Development, Abbott Nutrition, 18004 Granada, Spain; ricardo.rueda@abbott.com; 6 Research and Development, Abbott Nutrition, Columbus, OH 43219, USA; suzette.pereira@abbott.com

**Keywords:** curcumin, blood flow, glucose metabolism, skeletal muscle, ageing

## Abstract

Therapeutic interventions aimed at enhancing blood flow may combat the postprandial vascular and metabolic dysfunction that manifests with chronological ageing. We compared the effects of acute curcumin (1000 mg) coupled with an oral nutritional supplement (ONS, 7.5 g protein, 24 g carbohydrate and 6 g fat) versus a placebo and ONS (control) on cerebral and leg macrovascular blood flow, leg muscle microvascular blood flow, brachial artery endothelial function, and leg insulin and glucose responses in healthy older adults (*n* = 12, 50% male, 73 ± 1 year). Curcumin enhanced m. tibialis anterior microvascular blood volume (MBV) at 180 and 240 min following the ONS (baseline: 1.0 vs. 180 min: 1.08 ± 0.02, *p* = 0.01 vs. 240 min: 1.08 ± 0.03, *p* = 0.01), and MBV was significantly higher compared with the control at both time points (*p* < 0.05). MBV increased from baseline in the m. vastus lateralis at 240 min after the ONS in both groups (*p* < 0.05), and there were no significant differences between groups. Following the ONS, leg blood flow and leg vascular conductance increased, and leg vascular resistance decreased similarly in both conditions (*p* < 0.05). Brachial artery flow-mediated dilation and middle cerebral artery blood flow were unchanged in both conditions (*p* > 0.05). Similarly, the curcumin and control groups demonstrated comparable increases in glucose uptake and insulin in response to the ONS. Thus, acute curcumin supplementation enhanced ONS-induced increases in m. tibialis anterior MBV without potentiating m. vastus lateralis MBV, muscle glucose uptake, or systemic endothelial or macrovascular function in healthy older adults.

## 1. Introduction

Chronological ageing is a key risk factor for cardiovascular disease, which is the primary cause of mortality in developed societies [1,2,3]. The manifestation of endothelial dysfunction and arterial stiffness with ageing is a primary contributor to increased cardiovascular disease risk [4]. Additionally, both microvascular (i.e., resistance artery) and macrovascular (i.e., conduit artery) endothelial function, which each independently predict cardiovascular disease and mortality risk [5,6,7], decline with age. This inevitable and ensuing reduction in ageing muscle perfusion is a central tenet in the etiology of sarcopenia (age-related muscle mass and functional decline [8]), which increases the risk of frailty [9], morbidity [10], and mortality [11]. As such, effective therapeutic interventions that mitigate age-related vascular dysfunction are necessary to reduce the risk of cardiovascular and metabolic decline in the current ageing population [12].

While a variety of nutritional interventions have been shown to impact vascular responses [13,14], polyphenols have gained traction in recent years due to their antioxidant [15] and anti-inflammatory [16,17] properties, which provide therapeutic benefits in many noncommunicable diseases including, but not limited to, cancer, neurodegenerative disease, and diabetes [18]. The polyphenol curcumin favorably impacts skeletal muscle, attenuating muscle damage [17,19], and muscle atrophy [20], and enhancing exercise performance [17,21], muscle recovery/regeneration [17,19], and mitochondrial function and biogenesis [22]. These beneficial effects of curcumin may be perfusion-mediated as chronic (i.e., repeated) curcumin supplementation beneficially impacts endothelial function in humans [23]. Supporting this suggestion as well as confirming earlier preclinical findings [24], chronic curcumin supplementation improved resistance artery endothelial function, via reducing oxidative stress and increasing vascular nitric oxide bioavailability, and enhanced macrovascular endothelial function in both middle- and older-aged adults [12].

Curcumin’s purported vascular effects are not isolated to muscle as it also has systemic endothelial benefits [12,25,26]. Despite largely positive findings, to the best of our knowledge the impact of acute curcumin supplementation has been scarcely studied. The results from one study suggested that acute supplementation of curcumin-containing curry may improve flow-mediated dilation (FMD), a measure of endothelial function, in middle-aged healthy males [26]. However, because this study delivered curcumin via a curry meal that contained additional polyphenols, it is possible that the beneficial physiological outcomes were mediated, at least in part, by other polyphenols and not just curcumin. Because of the absence of other available studies, the isolated effects of acute curcumin, especially in older adults, remains poorly defined. Furthermore, curcumin-mediated systemic vascular benefits may translate into improved cerebrovascular function, via the enhanced delivery of oxygen and nutrients to the brain, which may help offset age-related cerebral blood flow decline and subsequent cognitive decline [27,28]. While one previous study found that chronic curcumin supplementation had no impact on cerebrovascular function in older overweight and obese adults [27], its impact in healthy older adults following acute supplementation remains to be investigated.

Curcumin also demonstrates antidiabetic properties [29]. For example, curcumin lowered the number of prediabetics progressing to type II diabetes [29], which may have been due to curcumin-enhancing vascular actions that increased nutrient and oxygen delivery to muscles. Considering that insulin resistance manifests with advancing age and contributes to the development of sarcopenia [30], curcumin may significantly benefit human health if it elicits synergistic cardiometabolic improvements. Curcumin supplementation has been shown to improve the glucose profile in patients with nonalcoholic fatty liver disease [31] and type II diabetes [32,33], and it improves glucose uptake [34] and insulin sensitivity [35] via AMPK signaling in preclinical models. However, the temporal relationship between vascular and metabolic responses following curcumin exposure remains to be determined in older adults.

As such, it is plausible to hypothesize that acute curcumin supplementation may favorably impact aspects of both limb and brain perfusion, which may translate into improved glucose handling. Thus, the aim of this study was to investigate the efficacy of acute curcumin supplementation on enhancing oral-feeding-induced changes in macrovascular (limb) blood flow, microvascular blood flow of the m. vastus lateralis and m. tibialis anterior, endothelial function, cerebral blood flow, and metabolic responses in healthy older adults.

## 2. Materials and Methods

### 2.1. Ethical Approval

The risks and procedures of this study were thoroughly explained to volunteers prior to obtaining written informed consent. The University of Nottingham Faculty of Medicine and Health Sciences Research Ethics Committee (2-1704) reviewed and approved the study, which was conducted in accordance with the Declaration of Helsinki [36]. This study was preregistered on clinicaltrials.gov (accessed on 6 February 2022) (NCT03213340).

### 2.2. Volunteers and Study Design

For this crossover, single-blind, placebo-controlled, randomized trial, healthy older adults (≥65 years, body mass index 18–30 kg/m^2^) were recruited via an internal recruitment database and from the local community. During an initial screening visit, volunteers were deemed eligible if they had a normal blood profile (complete blood count; liver and kidney function; HbA1c < 6%), were free from active metabolic disease, had a blood pressure of <160/100 mmHg, and were able to provide written informed consent. Volunteers were deemed ineligible if they had: renal, inflammatory bowel, cardiovascular, or cerebrovascular disease; active malignancy; clotting dysfunction; familial history of premature mortality (<55 years) from cardiovascular disease; a history of deep vein thrombosis; or a history of significant neurological or musculoskeletal disorders. Volunteers were also deemed ineligible if they reported: smoking; taking beta-adrenergic blocking agents; taking curcumin-containing supplements; taking part in regular or strenuous (>1x/week) exercise; a known intolerance to Sonovue or any of the study supplements; having recent surgery (within the previous 3 months); or being unable to adhere to the study protocol. During the screening visit, volunteers also performed a handgrip strength test and a short physical performance battery test.

**Figure 1:**
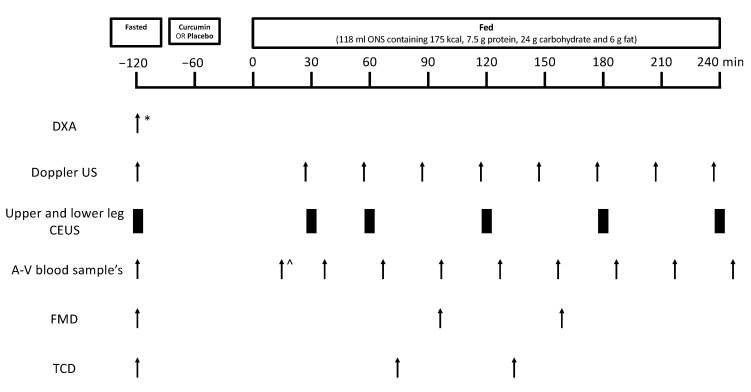
Study protocol schematic. The * indicates assessment was carried out during study visit 1 only, ^ indicates that the first blood draw occurred 15 min after the oral nutritional supplement. Arrows and black rectangles indicate when assessments were carried out. Abbreviations: A-V, arteriovenous; CEUS, contrast-enhanced ultrasound; DXA, dual-energy X-ray absorptiometry; FMD, flow-mediated dilation; TCD, transcranial Doppler; US, ultrasound.

Eligible volunteers participated in two experimental study visits separated by a “wash-out” period of 10–15 days. Volunteers were instructed to refrain from heavy exercise in the 48 h prior to study visits and were asked to refrain from taking medications that could alter blood flow (e.g., decongestants and angiotensin-converting enzyme inhibitors) on the day prior to and on the day of study visits. Volunteers arrived fasted to each study visit, defined as having no food or drink except water from 10 pm the previous evening, and had microvascular blood flow (MBF) measured via contrast-enhanced ultrasound (CEUS), leg blood flow (LBF) measured via Doppler ultrasound, endothelial function measured via FMD, middle cerebral artery blood flow velocity measured via transcranial Doppler (TCD), and a baseline blood sample taken. In addition, on study visit 1 only, dual X-ray absorptiometry (DXA; Luna Prodigy II; GE Medical Systems, Little Chalfont, Buckinghamshire, UK) was used to measure lean leg mass. A Philips iU22 ultrasound machine (Philips Healthcare, Reigate, Surrey, UK) was used for all ultrasound measures. Thereafter, the study supplement (curcumin or placebo) was orally consumed by the volunteers followed by an oral nutritional supplement (ONS). Doppler ultrasound, CEUS, FMD, and TCD measurements and blood samples were periodically obtained over the subsequent 4 h time frame (Figure 1).

### 2.3. ONS Feeding and Study Supplements

In a crossover design, volunteers randomly received curcumin or placebo. The curcumin supplement comprised 2 capsules, each containing 500 mg of formulated curcumin (solid lipid curcumin particles, Longvida, Verdure, Noblesville, IN, USA), delivering a total of 1000 mg curcumin (containing ~250–280 mg curcuminoids). Smaller doses of solid lipid curcumin particles (650 mg) have demonstrated good plasma bioavailability (~22 ng/mL) in healthy humans [37], so it was hypothesized that the higher dose of 1000 mg would be enough to elicit physiological effects. The placebo condition comprised two empty capsules matched for appearance.

Exactly 60 min after consuming curcumin or placebo, volunteers consumed an ONS (Ensure Advance (Vanilla), Abbott Nutrition, Hoofddorp, The Netherlands). This ONS was provided as it contains mixed macronutrients (175 kcal, 7.5 g protein, 24 g carbohydrate, and 6 g fat), which are known to stimulate vascular responses [14,38,39]. Considering the mean peak plasma curcumin response has been shown to occur 2 h following solid lipid curcumin particles [37], we speculated that the administration of solid lipid curcumin particles 60 min before the ONS would allow the peak plasma curcumin levels to coincide with the peak plasma insulin response to the ONS. Thus, this protocol allowed us to investigate whether curcumin enhances vascular and metabolic responses beyond those of a mixed meal.

### 2.4. Measurement of MBF Using CEUS

CEUS permits the measurement MBF and its components, microvascular blood volume (MBV) and microvascular flow velocity (MFV) and was described in detail previously [40]. In brief, Sonovue microbubbles (Bracco, Milan, Italy) infused via an antecubital fossa vein were detected via ultrasound. A linear probe was positioned on the m. tibialis anterior and on the m. vastus lateralis to detect intravascular microbubble concentration in the muscles. To disrupt microbubbles, intermittent high mechanical index “flashes” were used, with subsequent continuous low mechanical index recording measuring the rate of microbubble reappearance after each flash. Initially, Sonovue was infused at 2 mL/min for 1 min and then 1 mL/min for 3 min thereafter. In total, Sonovue was infused for 4 min. At 2.5 min, 3 30 s flash/replenishment recordings were made across the last 90 s of the protocol at each CEUS time point. After each flash, a 0.48 s window was used to adjust for noncontrast signal and for rapid filling of larger conduit vessels. The acoustic intensity of insonated tissue in the postflash period demonstrates a first-order exponential association function with a rate constant that is proportional to MFV and a plateau proportional to MBV. During CEUS measurements (<10 min), volunteers were asked to remain quiet and still.

### 2.5. Measurement of LBF Using Doppler Ultrasound

Using Doppler ultrasound, LBF was measured as previously described [41]. Herein, a L17-5 mHz probe was placed over the left common femoral artery to facilitate the assessment of LBF as vessel cross-sectional area x mean velocity over 6 cardiac cycles. To enhance the ultrasound signal, measurements were taken using ultrasound gel, with three measurements conducted at each time point. During the measurement period, volunteers were free of aural and visual stimuli and remained in a supine position. Leg vascular conductance (LVC) was calculated as: LBF/mean arterial pressure (which was calculated as: (2/3 diastolic blood pressure) + (1/3 systolic blood pressure)). Leg vascular resistance (LVR) was calculated as: mean arterial pressure/LBF [38,39,42,43]. LBF was adjusted to lean leg mass and standardized to fasting LBF.

### 2.6. Measurement of Systemic Endothelial Function and Cerebrovascular Function

FMD was used to assess brachial artery endothelial function using standard methodology [44]. Using a 17-5 MHz linear probe on the volunteers’ right arm after a baseline measurement of brachial artery diameter for 1 min, arterial occlusion distal to the brachial artery was induced using a blood pressure cuff (Hokanson, WA, USA) inflated to 200 mmHg for 5 min. Thereafter, the cuff was deflated, and dilation of the brachial artery was assessed for a subsequent 5 min. Quipu Cardiovascular Suite FMD Studio (Quipu, Tuscany) was used to generate automated real-time arterial diameter measurements. Due to technical failure, FMD was not recorded for 4 volunteers (data are *n* = 8).

Using standard techniques [45,46], TCD ultrasonography was used to measure middle cerebral artery blood flow velocity as an index of cerebrovascular function [47]. In brief, a 5-1 MHz probe was used to locate the transtemporal window needed to measure middle cerebral artery blood flow velocity. All measurements were performed by the same technician, and depths of insonation (between 50–60 mm) were recorded so they could be duplicated in each volunteer’s second study visit. It was not possible to locate the transtemporal window in 1 volunteer (data are *n* = 11).

### 2.7. Blood Sampling

The arteriovenous (A-V) sampling approach was used to assess glucose uptake and release. To facilitate this approach, blood glucose concentrations across the leg were measured (Glucose Analyzer, YSI, Yellow Springs) by sampling venous (using the Fick principle) and arterial bloods (obtained via the “hot-hand” technique) [48,49]. Plasma insulin concentrations were measured using a high-sensitivity human insulin enzyme-linked immunosorbent assay (DRG Instruments GmbH, Marburg, Germany). Blood samples were collected at baseline and 15, 35, 65, 95, 125, 155, 185, 215, and 245 min following ONS consumption.

### 2.8. Statistical Analysis

To determine supplement x time effects, two-way repeated measures ANOVA with Sidak’s and Dunnett’s multiple comparison analyses was conducted. Insulin and blood glucose A-V balance data were subjected to area under the curve (AUC) analysis, with paired *t*-tests used to determine supplement effects. Data were accepted as significant if *p <* 0.05. Data are presented as mean ± SEM. All data analysis was conducted using GraphPad Prism version 8 (GraphPad Software, San Diego, CA, USA).

## 3. Results

### 3.1. Volunteer Characteristics

Twelve healthy older males (*n* = 6) and females (*n* = 6) completed this trial, with volunteer baseline characteristics displayed in Table 1. No adverse events were reported in response to curcumin or placebo supplementation, indicating that curcumin was well-tolerated by volunteers.

### 3.2. Microvascular Responses to Curcumin Supplementation

**Figure 2:**
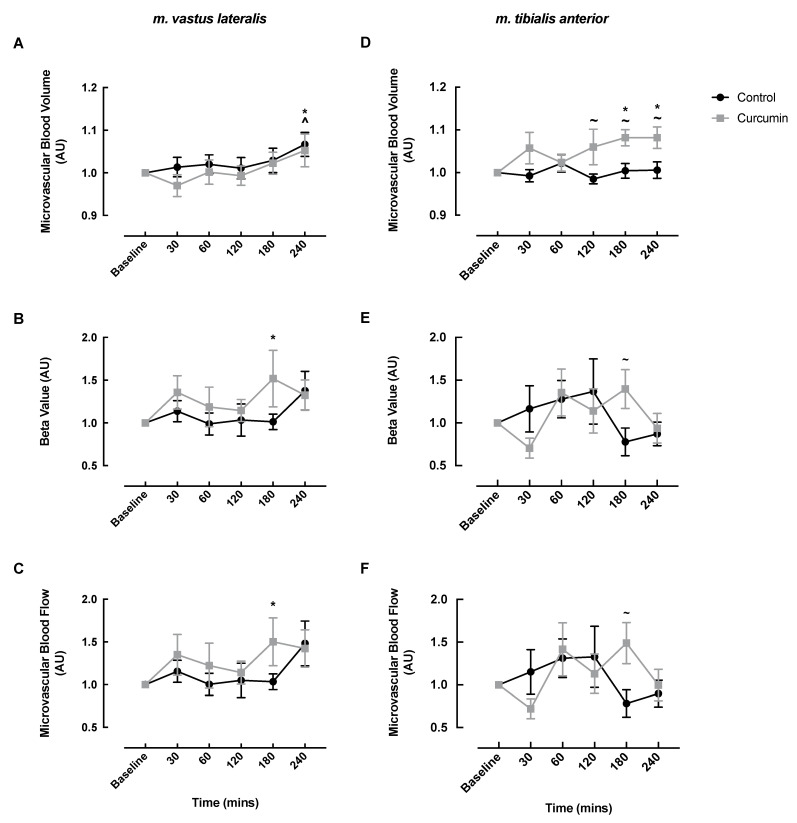
The impact of curcumin alongside oral nutritional supplement feeding on microvascular blood volume (**A**,**D**), microvascular flow velocity (**B**,**E**), and microvascular blood flow (**C**,**F**) in the m. vastus lateralis (**A**–**C**) and m. tibialis anterior (**D**–**F**) of healthy older adults. A ~ denotes a significant difference between groups (*p* < 0.05); ^ denotes significant difference from control baseline (*p* < 0.05); * denotes significant difference from curcumin baseline (*p* < 0.05).

In the m. vastus lateralis, MBV responses to the ONS significantly increased at 240 min in both the curcumin and control conditions (curcumin baseline: 1.0 vs. 240 min: 1.05 ± 0.04, *p* = 0.026; control baseline: 1.0 vs. 240 min: 1.07 ± 0.03, *p* = 0.003) (Figure 2A). MFV and MBF significantly increased at 180 min post-ONS in the curcumin condition only (MFV: baseline: 1.0 vs. 180 min: 1.52 ± 0.33, *p* = 0.036; MBF: baseline: 1.0 vs. 180 min: 1.50 ± 0.28, *p* = 0.048) (Figure 2B,C).

In the m. tibialis anterior, MBV significantly increased from baseline in the curcumin condition at 180 and 240 min (baseline: 1.0 vs. 180 min: 1.08 ± 0.02, *p* = 0.011 vs. 240 min: 1.08 ± 0.03, *p* = 0.011) and was significantly greater compared with the control at 120 (control: 0.99 ± 0.01 vs. curcumin: 1.06 ± 0.04, *p* = 0.030), 180 (control: 1.00 ± 0.02 vs. curcumin: 1.08 ± 0.02, *p* = 0.023), and 240 min (control: 1.01 ± 0.02 vs. curcumin: 1.08 ± 0.03, *p* = 0.027) (Figure 2D). MFV and MBF were significantly greater in the curcumin condition compared with the control condition at 180 min (MFV: control: 0.78 ± 0.16 vs. curcumin: 1.40 ± 0.23, *p* = 0.033; MBF: control: 0.78 ± 0.16 vs. curcumin: 1.49 ± 0.24, *p* = 0.015), although neither treatment condition displayed significant changes from their respective baselines (Figure 2E,F).

### 3.3. Macrovascular Responses to Curcumin Supplementation

**Figure 3:**
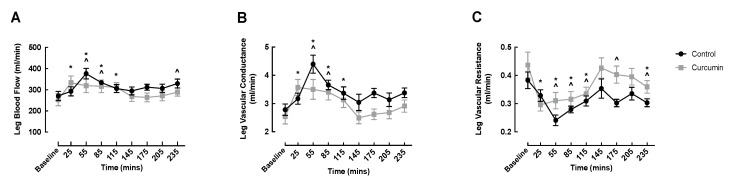
The impact of curcumin alongside oral nutritional supplement feeding on leg blood flow (**A**), vascular conductance (**B**), and vascular resistance (**C**) in healthy older adults. A ^ denotes significant difference from control baseline (*p* < 0.05); * denotes significant difference from curcumin baseline (*p* < 0.05).

LBF significantly increased from baseline in both conditions early in the fed phase (curcumin: baseline: 253.5 ± 28.5 vs. 25 min: 333.5 ± 31.5 mL/min, *p* = 0.001; control: baseline: 271.4 ± 22.0 vs. 55 min: 376.3 ± 25.3 mL/min, *p* < 0.0001), and the onset of increased LBF occurred earlier and remained elevated longer in the curcumin condition (Figure 3A). Similarly, LVC significantly increased from baseline in both conditions early in the fed phase, and curcumin supplementation evoked an earlier onset and prolonged rise in LVC (Figure 3B). LVR was depressed similarly in both groups, with significant declines observed at 55, 85, and 115 min (Figure 3C). After returning to basal values at 205 min, LVR was again depressed in both groups at 235 min (Figure 3C). No significant differences were detected between conditions at any time point for LBF, LVR, or LVR.

### 3.4. Endothelial and Cerebrovascular Responses to Curcumin Supplementation

**Figure 4:**
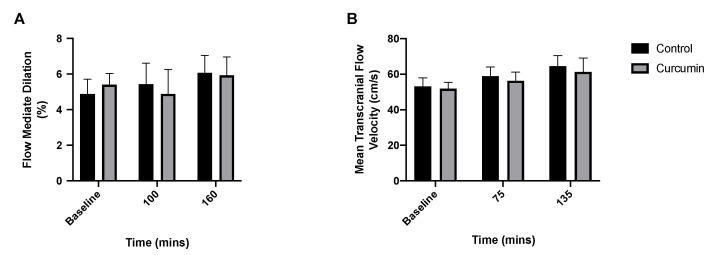
The impact of curcumin alongside oral nutritional supplement feeding on (**A**) flow-mediated dilation (*n* = 8 per condition) and (**B**) transcranial blood flow (*n* = 11 per condition) in healthy older adults.

Brachial artery FMD was unchanged from baseline in both curcumin and control conditions and was not different between conditions at any time point (*p* > 0.05) (Figure 4A). Similarly, middle cerebral artery blood flow was unchanged from baseline within conditions and was not different between conditions at any time point (*p* > 0.05) (Figure 4B).

### 3.5. Blood Glucose and Insulin Responses to Curcumin Supplementation

**Figure 5:**
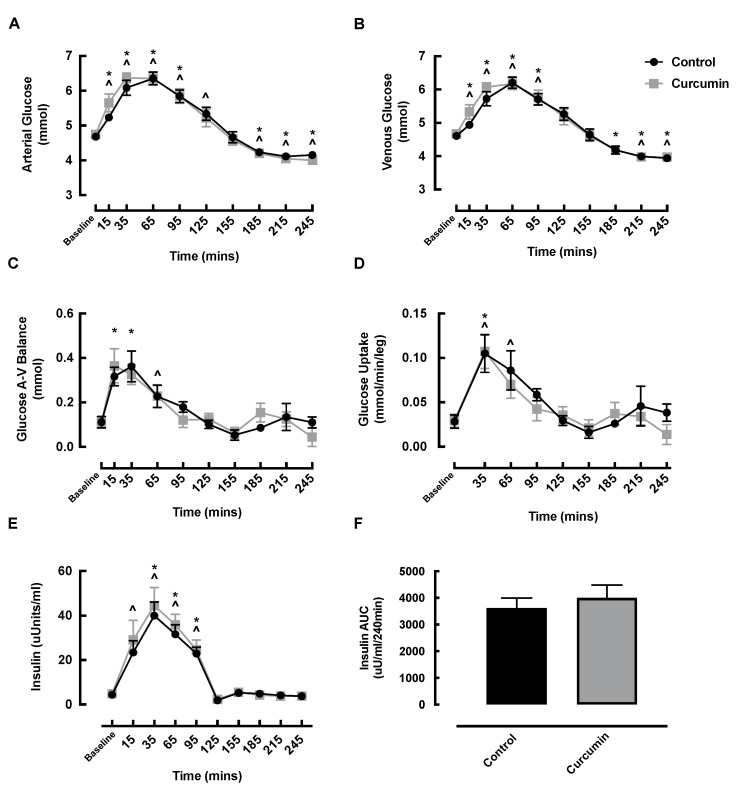
Effects of curcumin alongside oral nutritional supplement feeding on (**A**) arterial glucose; (**B**) venous glucose; (**C**) glucose A-V balance; (**D**) glucose uptake; (**E**) insulin; and (**F**) insulin area under the curve, in healthy older adults. ^ denotes significant difference from control baseline (*p* < 0.05); * denotes significant difference from curcumin baseline (*p* < 0.05). AUC, area under the curve; A-V, arteriovenous.

Arterial and venous glucose significantly increased in the early post-feeding phase in both the curcumin and control conditions (Figure 5A,B). Arterial glucose returned to baseline by 125 min in the curcumin condition, preceding the return to baseline in the control condition at 155 min (Figure 5A). Venous glucose returned to baseline by 125 min in both conditions (Figure 5B). No significant differences in arterial or venous glucose were observed between the conditions at any time point, and arterial and venous AUCs were not different between conditions (Figure S1).

Glucose A-V balance increased at 15 and 35 min in the curcumin condition (baseline: 0.11 ± 0.02 vs. 15 min: 0.36 ± 0.08 vs. 35 min: 0.33 ± 0.04 mmol, *p* < 0.05) and at 65 min in the control condition (baseline: 0.11 ± 0.03 vs. 65 min: 0.23 ± 0.05 mmol, *p* = 0.0004) compared with baseline; however, there were no significant differences between conditions for glucose A-V balance at any time point (Figure 5C) or for the glucose AUC (Figure S1). Glucose uptake increased at 35 min in the curcumin condition (baseline: 0.03 ± 0.01 vs. 35 min: 0.11 ± 0.02 mmol/min/leg, *p* = 0.005) and at 35 and 65 min in the control condition (baseline: 0.03 ± 0.01 vs. 35 min: 0.10 ± 0.02 vs. 65 min: 0.09 ± 0.02 mmol/min/leg, *p* < 0.05); however, there was no significant difference between conditions at any time point for glucose uptake (Figure 5D) or the glucose uptake AUC (Figure S1).

Following the ONS, insulin significantly increased at 15 min and remained elevated at 95 min in the control condition, whereas the curcumin condition displayed a slightly later onset of insulin responses, displaying a significant increase at 35 min that remained elevated at 95 min (Figure 5E). However, there was no significant difference between the conditions at any time point in insulin (Figure 5E) or for the insulin AUC (Figure 5F).

## 4. Discussion

Vascular function-enhancing interventions, such as polyphenols, may ameliorate age-related vascular and metabolic dysfunction. Although plenty of evidence has been published showing the benefit of chronic curcumin supplementation [23,25], it is not known if these bioactives have an acute effect on blood flow. Hence, we investigated the acute impact of a bioavailable curcumin extract [37], concurrent with an oral mixed-macronutrient meal, on vascular and subsequent metabolic responses in healthy older adults. We found acute curcumin supplementation (1000 mg, containing ~250–280 mg curcuminoids) enhanced MBV in the m. tibialis anterior but not the m. vastus lateralis beyond the effect of a small mixed-macronutrient meal, but it did not influence systemic endothelial or cerebrovascular function. Notably, enhanced MBV did not translate into improved insulin or glucose responses.

We chose to measure microvascular blood flow in both the m. tibialis anterior and m. vastus lateralis as they represent phenotypically different muscles, and thus it is plausible that curcumin may have divergent impacts in either muscle. For example, compared to the m. vastus lateralis, the m. tibialis anterior is a smaller, more oxidative, and capillary-dense muscle [50], which is composed of ~70% type I fibres [51]. Our primary finding was that an acute dose of curcumin enhanced the effect of a small mixed-macronutrient meal by increasing MBV in the m. tibialis anterior of older healthy men and women. This finding suggests that curcumin impacts the vasodilation of the muscle capillaries, driving blood and oxygen and nutrients into the m. tibialis anterior beyond the effects of the postprandial insulin response. Although the mechanisms of action were not probed, chronic curcumin supplementation was previously shown to enhance vascular function in a similar population by reducing vascular oxidative stress and increasing nitric oxide availability [12]; thus, that is a possible mechanism underlying the vascular response we observed. Curcumin did not potentiate feeding-induced MBV increases in the larger m. vastus lateralis muscle. There is no clear explanation for this finding, especially considering that larger muscles have a larger region of interest available for CEUS analysis, which should lead to less technical variation and make statistical significance easier to reach. In fact, earlier studies showed that certain plant phenols (e.g., cocoa flavanols and green tea extract) are able to enhance feeding-induced MBV of the m. vastus lateralis [38,39]. As such, these findings must be verified in larger clinical trial cohorts before drawing any firm conclusions regarding the muscle-type-specific effects of curcumin.

An enhancement in m. tibialis anterior microvascular perfusion did not translate into improved insulin or glucose responses. Outwardly, this may seem somewhat surprising as MBV is an important precursor and potential rate-limiting step for insulin-mediated glucose disposal [52], so potentiated MBV may be expected to translate to enhanced leg glucose uptake. Additionally, previous studies have shown that chronic curcumin supplementation improves insulin resistance in type II diabetics [53] and lowers blood glucose levels in people with dysglycemia [54], indicating that curcumin favorably impacts one’s metabolic profile, which is potentially mediated via enhanced perfusion. However, it is also possible that more-sensitive methods than those used in this study (e.g., glucose A-V balance) are needed to detect small yet significant localized changes, such as those of glucose uptake into muscle, e.g., using 2-deoxyglucose [55]. Nonetheless, our data suggest that glucose uptake is primarily driven by the insulin-mediated impact of mixed feeding and is not further enhanced by curcumin. Insulin and glucose kinetics were also similarly regulated between the curcumin and control groups, further supporting the effects of the mixed meal. It is also possible that the lack of alignment in MBV responses in the different muscle groups underlay the lack of, or mask changes in, insulin and glucose kinetics. Therefore, the precise physiological relevance of enhanced late muscle perfusion following acute curcumin remains to be fully deciphered but likely does not mediate transient anabolism (in line with our previous data [41,56]) or leg glucose uptake in healthy older adults.

Despite enhanced MBV (in the m. tibialis anterior), curcumin did not further impact macrovascular blood flow (LBF, LVC, and LVR) beyond the observed modulation by the ONS, demonstrating that macrovascular femoral artery flow was driven by the insulin response to the mixed meal and was not further enhanced by curcumin. It is likely that longer term supplementation is needed to observe the impact of curcumin on macrovascular flow, as other studies have shown that chronic curcumin supplementation positively impacts macrovascular flow outcomes in young [57], middle-aged, and older adults [12].

In addition to local vascular effects (i.e., muscle effects), curcumin has purported systemic vascular benefits [12,25,26]. Using FMD, we found that acute curcumin supplementation did not impact systemic endothelial function in older adults. In line with our data, Barber-Chamoux et al. found that acute curcumin supplementation (5 g) in smokers did not improve FMD [58]. However, subgroup analyses revealed that FMD was mediated by curcumin in females and in those with lower cardiovascular risk [58]. Conversely, our data are in disagreement with those of others who found that acute oral supplementation of curcumin-containing curry improved FMD [26]. However, in the study in question [26], the curry spice tested contained additional polyphenols that may have mediated FMD responses. More recently, a meta-analysis concluded that curcumin increases FMD [25]. However, only five clinical trials were included in this analysis, and among these five studies, age and supplementation regime were varied (among other things such as curcumin formulation), precluding robust age- and dosing-regimen-specific conclusions. Nonetheless, further acute supplementation studies are required in older adults to clarify the links between curcumin and systemic endothelial function.

Whether curcumin’s beneficial effects on perfusion translates to improved cerebrovascular function via optimal oxygen and nutrient delivery to the brain [27] remains an important yet poorly defined question. This is particularly relevant for older adults as cerebral blood flow declines with ageing and may manifest as cognitive decline [27,28]. We found that curcumin had no impact on cerebrovascular function measured by TCD, which may be explained by the lack of a systemic endothelial function response. In line with our data, Kuszewski et al. [27] found no impact of (chronic) curcumin supplementation on cerebrovascular function in older sedentary overweight and obese adults. As such, curcumin dose–response studies are necessary to identify the optimal curcumin dose for mediating cerebrovascular responses.

We acknowledge that the absence of curcumin bioavailability data is a limitation of this study. However, others have reported good bioavailability with smaller doses of curcumin. For example, Gota and colleagues reported a mean peak plasma curcumin concentration of ~22ng/mL 2 h after healthy volunteers consumed 650 mg of solid lipid curcumin particles [37]. Additionally, this solid lipid curcumin particle-based formula demonstrated superior bioavailability when compared with an equal amount of unformulated 95% curcuminoids [37]. Furthermore, the solid lipid curcumin particle complex (i.e., the curcumin formulation provided herein) is purported to prevent rapid curcumin degradation and excretion, thereby enhancing systemic curcumin concentration and half-life [37,59]. Collectively, this suggests that the 1000 mg dose of curcumin provided herein was bioavailable. We only investigated the effects of one dose of curcumin (1000 mg), precluding any interpretation into the hormetic properties of curcumin in healthy older adults. Indeed, preclinical and diseased cohorts demonstrate that curcumin acts in a hormetic-like manner. Thus, there is a need for future research to comprehensively investigate the dose–response relationship of curcumin to ensure that desired physiological responses are achieved [60].

## 5. Conclusions

Acute curcumin supplementation potentiated MBV in the m. tibialis anterior beyond the effect of a small mixed meal but did not improve systemic endothelial or cerebrovascular function. Enhanced MBV did not, however, translate to improved leg glucose uptake. Whether the vascular effects of curcumin are truly muscle-specific requires validation in larger clinical trials before robust conclusions can be drawn regarding the efficacy of curcumin for optimizing muscle vascular responses in healthy older adults.

## Figures and Tables

**Table 1:** Volunteer baseline characteristics (mean ± SEM).

Parameter	Volunteers (*n* = 12)
Gender (% M)	50
Age (years)	73 ± 1
Height (cm)	171.5 ± 2.8
Body mass (kg)	79.4 ± 4.4
BMI (kg/m^2^)	26.7 ± 0.8
Lean mass (kg)	50.0 ± 3.5
Resting heart rate (bpm)	62 ± 2
Resting systolic blood pressure (mmHg)	137 ± 3
Resting diastolic blood pressure (mmHg)	79 ± 3
Grip strength (kg)	29.7 ± 2
SPPB	11 ± 0.3

BMI, body mass index; SPPB, short physical performance battery.

## Data Availability

The data presented in this study are available upon reasonable request from the corresponding author.

## Supplementary Materials

The following supporting information is available at: https://www.mdpi.com/article/10.3390/nu14061313/s1, Figure S1: Arterial glucose (A), venous glucose (B), glucose arteriovenous balance (C), and glucose uptake (D) area under the curve in healthy older adults, with and without curcumin, following oral nutritional supplement feeding. AUC, area under the curve; A-V, arteriovenous.
