# Supplementary material for: Curcumin Enhances Fed-State Muscle Microvascular Perfusion but Not Leg Glucose Uptake in Older Adults
Source: Nutrients. 2022 Mar 21;14(6):1313. doi: 10.3390/nu14061313 (PMC8953570; doi:10.3390/nu14061313)
Supplement: Supplementary file 1 [file nutrients-14-01313-s001.zip › nutrients-1608102-supplementary.pdf]

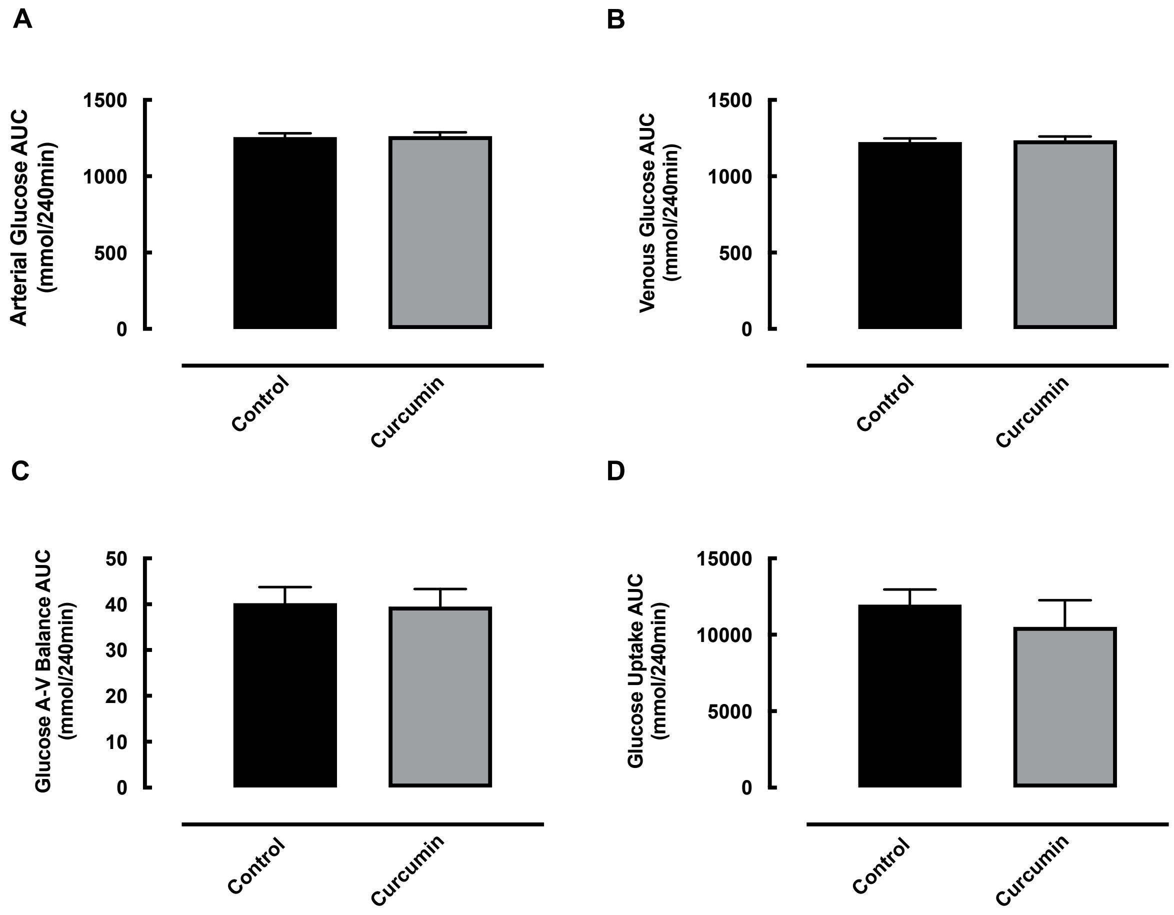

Figure S1. Arterial glucose (A), venous glucose (B), glucose arterio-venous balance (C) and glucose uptake (D) area under the curve in healthy older adults with/without curcumin, following oral nutritional supplement feeding. AUC, area under the curve; A-V, arterio-venous.
